# Functional diversification of Argonautes in nematodes: an expanding universe

**DOI:** 10.1042/BST20130086

**Published:** 2013-07-18

**Authors:** Amy H. Buck, Mark Blaxter

**Affiliations:** Centre for Immunity, Infection and Evolution, Ashworth Laboratories, University of Edinburgh, West Mains Road, Edinburgh EH9 3JT, U.K.

**Keywords:** Argonaute, helminth, microRNA (miRNA), nematode, RNA interference (RNAi), small interfering RNA (siRNA), Ago, Argonaute, ALG, Ago-like gene, *At*, *Arabidopsis thaliana*, CSR, chromosome segregation- and RNAi-deficient, miRNA, microRNA, piRNA, piwi-interacting RNA, PRG, Piwi-related gene, RdRP, RNA-dependent RNA polymerase, RDE, RNAi-defective, RISC, RNA-induced silencing complex, RNAi, RNA interference, siRNA, small interfering RNA, WAGO, worm-specific Ago

## Abstract

In the last decade, many diverse RNAi (RNA interference) pathways have been discovered that mediate gene silencing at epigenetic, transcriptional and post-transcriptional levels. The diversity of RNAi pathways is inherently linked to the evolution of Ago (Argonaute) proteins, the central protein component of RISCs (RNA-induced silencing complexes). An increasing number of diverse Agos have been identified in different species. The functions of most of these proteins are not yet known, but they are generally assumed to play roles in development, genome stability and/or protection against viruses. Recent research in the nematode *Caenorhabditis elegans* has expanded the breadth of RNAi functions to include transgenerational epigenetic memory and, possibly, environmental sensing. These functions are inherently linked to the production of secondary siRNAs (small interfering RNAs) that bind to members of a clade of WAGOs (worm-specific Agos). In the present article, we review briefly what is known about the evolution and function of Ago proteins in eukaryotes, including the expansion of WAGOs in nematodes. We postulate that the rapid evolution of WAGOs enables the exceptional functional plasticity of nematodes, including their capacity for parasitism.

## Introduction

RNAi (RNA interference) was first described as the mechanism by which double-stranded RNA silences cognate sequences in nematodes [1], which paralleled earlier findings in plants showing that transgenes caused silencing of endogenous genes and this was mediated by RNA [2]. Since these discoveries, many versions of RNAi have been described in Eukarya, all of which involve RISCs (RNA-induced silencing complexes) minimally comprising one Ago (Argonaute) protein and one small RNA. There is extensive diversity in the functions of RISCs depending on the small RNA that is incorporated, the functional properties of the Ago and effector proteins, and the class of nucleic acid target (reviewed in [3]). Small RNAs incorporated into RISCs include miRNAs (microRNAs), endogenous or exogenous siRNAs (small interfering RNAs), piRNAs (piwi-interacting RNAs) and other transposon-associated small RNAs (reviewed in [4]); these differ in their origins, subcellular localizations and/or presence in different tissues.

Ago proteins have at least two important roles in RISCs: they must recognize and bind small RNAs and they must mediate interactions with other proteins required for loading small RNAs, association with targets, gene silencing activity and/or subcellular localization (reviewed in [5]). Some Agos can also participate in the biogenesis of small RNAs [6], but this does not appear to be a universal property. Given the central role of Agos in RISCs, the diversification of RNA-silencing functions is inherently linked to their evolution. In the present article, we summarize what is known about Ago evolution and function in eukaryotes, with a specific focus on emerging RNAi functions in nematodes.

## Argonaute proteins: structure and origin

Ago proteins are highly basic proteins approximately 90–100 kDa in size that contain at least two domains that are detectable from primary sequence analysis: the PAZ domain and the PIWI domain. The PAZ domain forms an OB (oligonucleotide/oligosaccharide-binding) fold that mediates interactions with the 3′-end of the small RNA. The PIWI domain folds into an RNaseH-like domain that, in some Agos, contains an active site for endonucleolytic cleavage of targets (termed ‘slicing’) (reviewed in [7,8]). Two additional domains are revealed in the crystal structures of full-length Agos: an N-terminal domain and a Mid-domain, which binds to the 5′-phosphate of the small RNA [9]. Ago proteins were originally grouped into two clades, Ago-like or Piwi-like, on the basis of similarity to either of two of the proteins first discovered: the AGO1 protein in *Arabidopsis thaliana* (*At*) [10] and the PIWI (P-element-induced wimpy testis) protein in *Drosophila melanogaster* [11]. There does not appear to be a universal difference in the mechanistic properties of Ago-like and Piwi-like proteins: proteins from both clades have been shown to function by transcript degradation or transcriptional silencing. For example, Ago-like proteins in plants can direct histone methylation (*At*Ago4) as well as endonucleolytic cleavage (*At*Ago1) [12–14]. Phylogenetic analyses suggest that both Ago-like and Piwi-like proteins were present in the last common ancestor of eukaryotes, but one or both have since been lost in specific lineages [15]. For example, fungi and plants only contain Ago-like proteins, whereas Amoebozoa and *Paramecium tetraurelia* contain only the Piwi-like proteins.

## Argonaute expansions in Eukarya

Genome defence is presumed to have been the ancestral function of RNAi and it remains a key feature of RNAi pathways across Eukarya [16]. However, additional functions have emerged in specific lineages. For example, the miRNA pathway arose early in the animal lineage and is essential for development and proposed to have enabled morphological complexity and tissue diversity [17]. miRNAs are also essential for development in plants and are assumed to have arisen independently [18]. Additional variants of the RNAi mechanism have been found in specific eukaryote lineages including RNAi-mediated (hetero)chromatin formation, programmed genome rearrangements (DNA elimination), meiotic silencing by unpaired DNA and RNA-directed DNA methylation [15].

Multiple factors could lend to the diversification of RNAi pathways, and it is likely that the expansion of Ago proteins through duplication is a key component of this diversification. In mouse and humans, there are four Ago-like proteins and four Piwi-like proteins. The Ago-like proteins probably evolved through duplications in vertebrates [19], but the importance of these duplications is not yet clear: only Ago2 has slicer activity, and the majority of miRNAs and targets appear to be shared among the four Agos [20]. Multiple Piwi-like proteins have been shown to play co-ordinated roles in sexual reproduction in both mouse and *D. melanogaster*, and their diversification may be important for spatial and temporal division of these functions [15]. Plant Agos are similarly diverse. There are ten Ago-like proteins in *A. thaliana*, which form three clades in phylogenetic analyses that have different substrates and targets, functioning as RNA slicers, RNA binders or chromatin modifiers [21,22]. Interestingly, one Ago from each clade is ubiquitous and highly expressed, whereas the additional Agos display tissue specificities indicative of roles in reproduction [21]. In *Oryza sativa* (rice), there are 18 Ago proteins, including four Ago1 homologues and two ‘orphan’ Agos that do not group with the others and whose functions are unknown [21].

At present, the greatest expansion of Ago proteins known is in the Nematoda: 25 Ago proteins are encoded in the *Caenorhabditis elegans* genome, of which 18 form a distinct clade of WAGOs (worm-specific Agos) that is distinct from Piwi-like and Ago-like clades [23]. Orthologues of these *C. elegans* WAGOs are found in other *Caenorhabditis* species (*C. briggsae*, *C. remanei* and *C. brenneri*) [24]. WAGOs are also present in more distantly related nematodes, such as *Ascaris suum* [25], and many of these can be classified within the orthology groups defined in *Caenorhabditis* species [26].

## WAGO functions in *C. elegans*

As in other animals, miRNAs, siRNAs and piRNAs direct RNAi pathways that are important for development, reproduction and genome defence in nematodes. However, unlike many animals, nematodes express RdRPs (RNA-dependent RNA polymerases) that serve to amplify RNAi responses. This is achieved through the production of ‘secondary’ siRNAs, which are generated by the recruitment of RdRPs to the nucleic acids targeted by Agos bound to a small ‘primary’ RNA [23,27,28]. The secondary siRNAs are the most abundant small RNA in *C. elegans*. They are generally 22 nt in length, and start with a 5′-terminal guanosine with a triphosphate, hence they have been named ‘22G RNAs’. The small primary RNAs that direct the location of siRNA synthesis on targets include piRNAs [29–31], exogenous dsRNA [32] and endogenous siRNAs including ‘26G RNAs’ (which themselves are derived from an RdRP) [33,34]. There is also an example of a miRNA acting as a guide for 22G RNA production when loaded into the Ago protein RDE1 (RNAi-defective 1) [35]. In line with the diversity of primary small RNA triggers, 22G RNAs can derive from, and subsequently target, a wide range of nucleic acids including protein coding genes, pseudogenes, transposons and non-annotated loci [23,33,35–39].

All 22G RNAs bind to members of the WAGO family and these specific associations direct their involvement in different pathways. For example, distinct subsets of 22G RNAs in the germline bind to WAGO1 or CSR-1 (chromosome segregation- and RNAi-deficient 1, another WAGO protein) [36]. The 22G RNAs that bind to WAGO1 target transposons, pseudogenes, aberrant transcripts or cryptic loci, and are proposed to function as a surveillance mechanism [36]. In contrast, the subset of 22G RNAs that bind to CSR-1 primarily derive from protein-coding genes. However, the targets of CSR-1-associated 22G RNAs are not silenced; instead, the 22G RNAs direct CSR-1 to protein-coding domains within chromatin, serving to organize them within holocentric chromosomes [40]. Recent reports suggest that 22G RNA pathways also direct multigenerational epigenetic memory in the germline: piRNAs and the Piwi-like protein PRG-1 (Piwi-related gene 1) direct the synthesis of 22G RNAs on foreign RNAs (and some endogenous RNAs). The mechanistic details of this process are still unfolding, but it requires both cytoplasmic and nuclear WAGOs as well as chromatin factors [29,41–43]. The piRNA/22G RNA pathway has therefore been proposed as a sophisticated mechanism for recognizing and silencing foreign sequences. Another 22G RNA pathway is likely to work in partnership with silencing of foreign sequences to maintain memory of ‘self’ RNA. The CSR-1/22G RNA pathway is a likely candidate for this self-recognition mechanism, since it marks protein-coding genes, but does not silence them [40–43].

From the above examples, it is clear that 22G RNA pathways and the associated WAGOs enable highly sophisticated and co-ordinated modes of genome defence and endogenous gene regulation. It seems likely that analyses in *C. elegans* have only scratched the surface of the modes of regulation that are possible and the mechanistic details that underpin them.

## Argonaute proteins in parasitic nematodes

**Figure 1:**
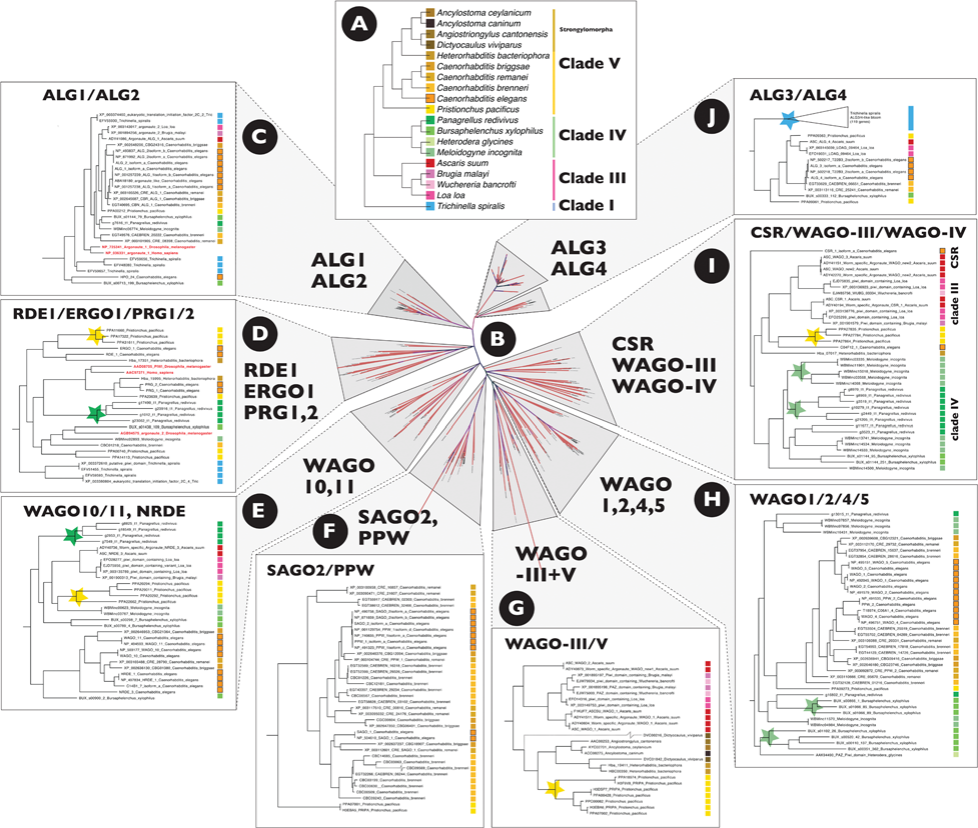
Phylogenetic analysis of Ago proteins from Nematoda (**A**) Cartoon of the phylogenetic relationships of the nematode species analysed, based on analyses of the nuclear small subunit ribosomal RNA gene. The colours associated with each species are used to decorate the subtrees in (**C**)–(**J**). Coloured stars on the subtrees indicate species-specific ‘blooms’ of paralogues. (**B**) The global tree of nematode Ago represented as an unrooted phylogram. We assume that each subtree is effectively rooted by the other subtrees, although we note that extreme divergence results in the support for structure within some subtrees being marginal. (**C**) The ALG1/ALG2 subtree, which contains representatives from all the nematode clades surveyed. (**D**) The RDE1/ERGO1 (endogenous RNAi-deficient Ago 1)/PRG1/PRG2 subtree, which contains no members from the animal parasites of Clade III. (**E**) The WAGO10/WAGO11/NRDE (nuclear RNAi-defective) subtree, which has wide representation across the Nematoda. (**F**) A SAGO2 (synthetic secondary siRNA-deficient Ago mutant 2)/PPW (PAZ/PIWI domain-containing) WAGO subtree, which is restricted to *Caenorhabditis* and *P. pacificus*. (**G**) A subtree of WAGO proteins, which lacks any members from *Caenorhabditis*, Clade IV or Clade I species, but is present in *P. pacificus* and animal parasites in Clades III and V. (**H**) The WAGO1/WAGO2/WAGO4/WAGO5 subtree, restricted to Clade IV and V nematode species, with a remarkable bloom of paralogues in *P. redivivus*. (**I**) This component of Ago diversity includes *C. elegans* CSR and a subtree of WAGO proteins restricted to Clade III, IV and V species, with a second paralogue bloom in *P. redivivus*. (**J**) The ALG3/ALG4 subtree, in which *T. spiralis* has a remarkable bloom of 119 distinct Ago proteins. Over 550 distinct Ago proteins (containing PIWI and PAZ domains) were obtained by extensive similarity searching of the NCBI NR protein database, WormBase nematode genome data (http://www.wormbase.org) and NEMBASE4 (http://www.nematodes.org), and aligned using CLUSTAL Omega. The alignment was analysed in MrBayes 3.2.1 using a mixed prior on amino acid evolution model, and run for 1 million generations. After visual inspection in Tracer (http://tree.bio.ed.ac.uk/software/tracer/), the first 500000 generations were discarded as burnin. The input sequences, alignment, MrBayes command block, treefiles and summary phylograms are available on DataDryad (doi:10.5061/dryad.5qs11). For improved clarity, a full-size PDF version of this Figure can be found at http://www.biochemsoctrans.org/bst/041/bst0410881add.htm.

Nematodes are extraordinarily abundant animals and show enormous functional diversity, including frequent acquisition of digestive system and tissue parasitism in a wide range of hosts, including all large-bodied animals and most plants. Animal parasitism has arisen at least six times independently in animals and three times in plants [43]. The nematode phylogeny reveals major clades where all members are parasitic (such as Clade III, and the Strongylomorpha within Clade V, Figure 1A). To understand the variety of small RNA pathways present in Nematoda, we have explored the diversity of Ago proteins in available transcriptome and genome data (Figure 1B). As expected, homologues of ALG1 (Ago-like gene 1) and ALG2, which bind to miRNAs, are present in all the fully sequenced nematode genomes (Figure 1C). Accordingly, miRNAs have been identified in all parasitic nematodes so far examined: *A. suum*, *Brugia malayi*, *Brugia pahangi*, *Dirofilaria immitis* (all in Clade III of the nematode phylogeny [43]), *Haemonchus contortus* (Clade V) and *Trichinella spiralis* (Clade I) [25,45–48] and the plant parasite *Bursaphelenchus xylophilus* (Clade IV) [49]. ALG3 and ALG4 homologues are also widely present (Figure 1J); these proteins are known to play a role in spermatogenesis [34]. Interestingly the Clade I parasite *Trichinella spiralis* has a striking idiosyncratic amplification of 119 ALG-like genes of unknown specific function.

On the basis of the absence of 21U RNAs in deep sequencing analyses, the Clade III parasites *A. suum* and *B. pahangi* appear to have lost the piRNA pathway [25,45]. Congruent with these observations, no homologues of the Piwi proteins PRG-1 or PRG-2 were detected in the *A. suum* or *B. malayi* genomes, and no PRG-like Ago genes are present in Clade III species surveyed (Figure 1D). It is not clear why the piRNA mechanism might be absent from Clade III species. This loss is not broadly linked with parasitism as piRNAs are present in the Clade V vertebrate gut parasites *H. contortus* [45] and *Heligmosomoides polygyrus* (A. Buck and R. Maizels, unpublished work).

A previous survey of proteins active in the RNAi machinery in parasitic nematodes showed that at least one RdRP was present in representatives of the nematode clades examined (Clades I, III, IV and V) [26]. Except for *Caenorhabditis* species, *A. suum* is the only nematode in which 22G RNAs have been characterized [25]. The lack of reports from other species may reflect the library preparation methods used (which did not capture 22G RNAs because of their 5′-triphosphate). From the phylogenetic analyses presented here, it is apparent that additional diverse WAGO genes are present in all the species analysed, across nematode diversity, which are separable into a number of radiations (Figure 1E–1I). It seems logical that these could play a number of functions, from genome defence to chromatin segregation to other functions required for sensing and adapting to a specific environment, in line with the recognition of foreign RNA proposed by Sarkies and Miska in this issue of *Biochemical Society Transactions* [50]. Given the utility of the RNA amplification mechanism, it seems likely that secondary siRNAs could be ubiquitous to WAGO pathways. However, whether these small RNAs are produced and operate by similar mechanisms in different nematodes remains to be determined. For example, in *A. suum*, 22G RNAs map across the length of their mRNA targets with increasing frequency at the 5′-end [25], whereas in *C. elegans*, they map to both termini [36]. This suggests differences in the biogenesis pathways active in these species. Similarly, a class of 26G RNA was identified in *A. suum* and *C. elegans*, where they play a role in spermatogenesis. However, the *C. elegans* and *A. suum* 26G RNAs must differ in their biogenesis, as *C. elegans* 26G RNAs are 3′-methylated, but *A. suum* 26G RNAs are not [25].

Despite the diversity of Ago-like genes in *C. elegans*, our phylogenetic analyses also suggest that some species may have lost distinct WAGO subtypes present in other nematodes. For example, a WAGO subtype present in strongylomorph and Clade III parasites, and the free-living Clade V species *Pristionchus pacificus*, has no representatives in any *Caenorhabditis* species (Figure 1G). Also striking is the presence in many species of idiosyncratic blooms of paralogous sequences, such as the *T. spiralis* ALG-like bloom mentioned above, and several sets of WAGO sequences with multiple closely related members in the free-living *Panagrellus redivivus* (marked with stars in Figure 1). The function of these proteins and the RNAs with which they interact requires further study, but it is tempting to speculate that they might be important for some aspect of the specific lifestyles of the species, including parasitism.

## Conclusions

Nematodes not only represent one of the pre-eminent, and revealing, model systems for understanding biology, but also are one of the most abundant and diverse of the animal phyla. They can survive in almost any habitat, including living as parasites within a wide phylogenetic range of hosts. Within the free-living *C. elegans*, and its relatives, a surprising diversity of small RNAs and small RNA-mediated biological regulation has become apparent, and this is necessarily accompanied by a diversity in small RNA protein partners, including the Ago proteins. The discovery of additional Ago diversity in parasitic nematode species raises the exciting possibility that these proteins may be involved in regulatory or sensing adaptations associated with parasitism: the challenges of finding and invading a host, and of establishing a niche within the host in the face of active immune responses.

Whereas RNAi is part of core innate immunity and has been co-opted into essential developmental roles, it is clear that natural selection will ensure that each organism is pressured to optimize its RNAi system to counter the particular challenges it faces [51]. These novel idiosyncratic applications of the RNAi pathway are not only revealing of the particular biology of the species in which they are found, but also may offer new routes to experimental manipulation of other species, or to development of specific therapies for damaging species. Defining molecular and physiological functions for the large number of diverse Ago proteins being discovered in nematodes is a challenge that is likely to be rewarding and revealing.

## Online data

Supplementary data
