# Supplementary figures and images for: Functional diversification of Argonautes in nematodes: an expanding universe
Source: Biochem Soc Trans. 2013 Jul 18;41(Pt 4):881–6. doi: 10.1042/BST20130086 (PMC3782831; doi:10.1042/BST20130086)

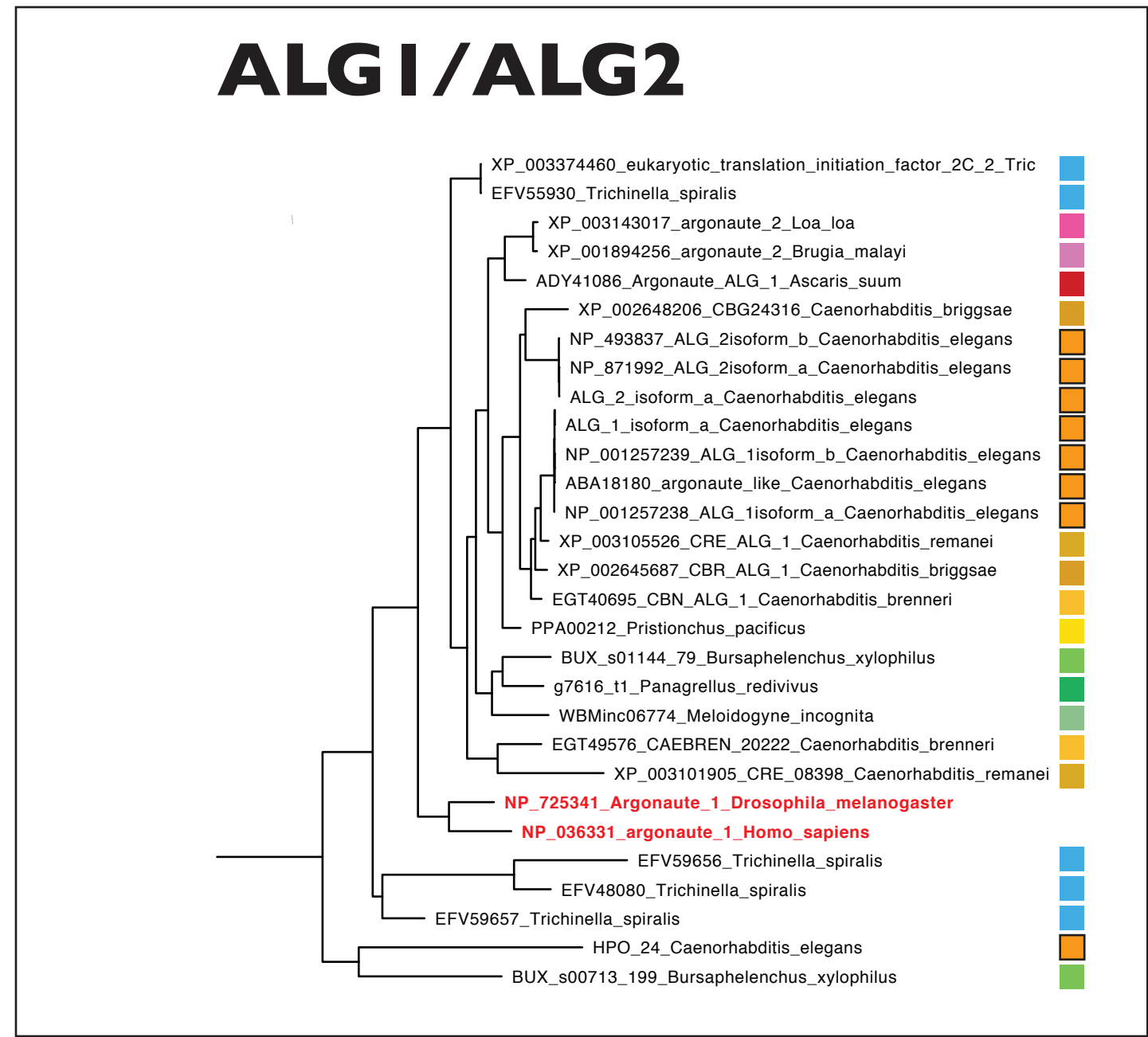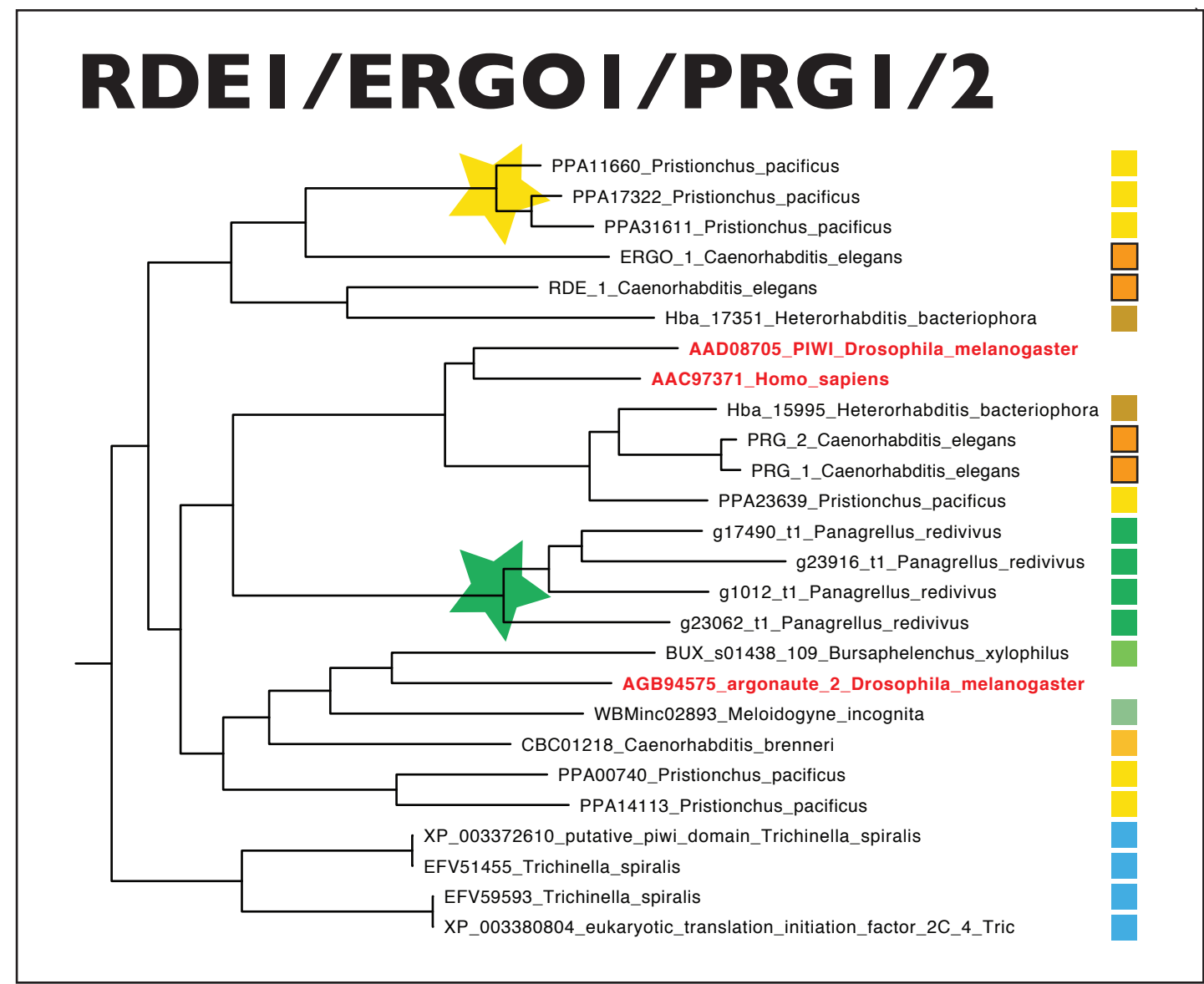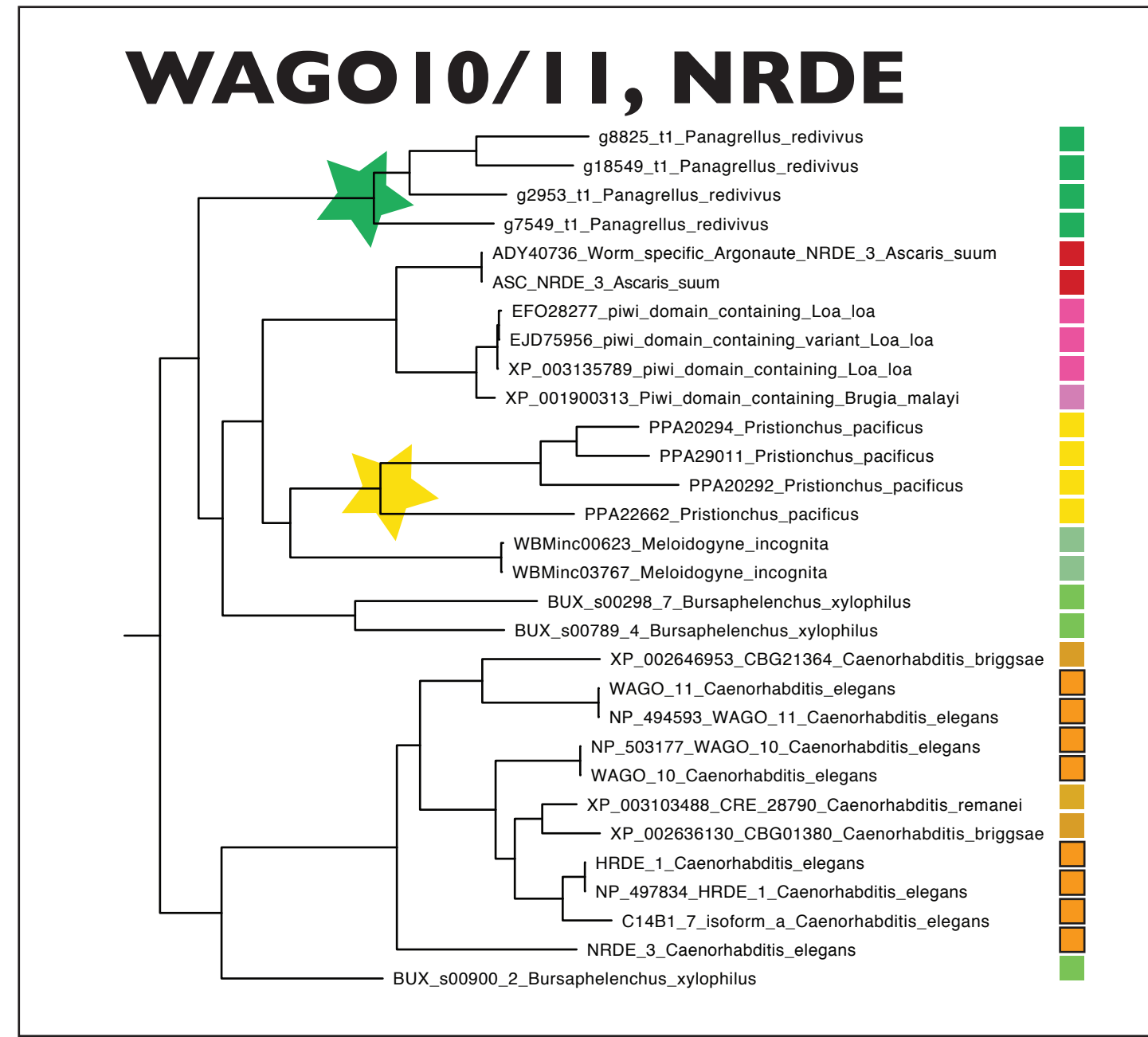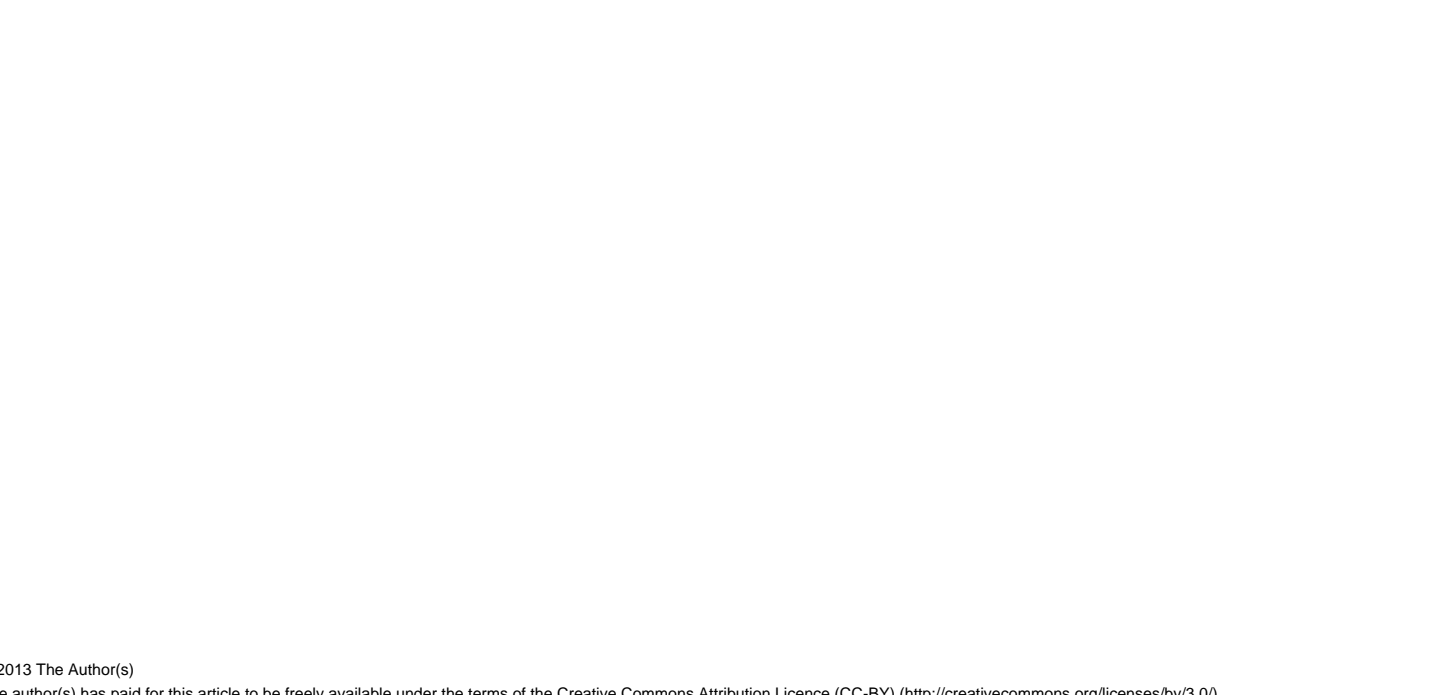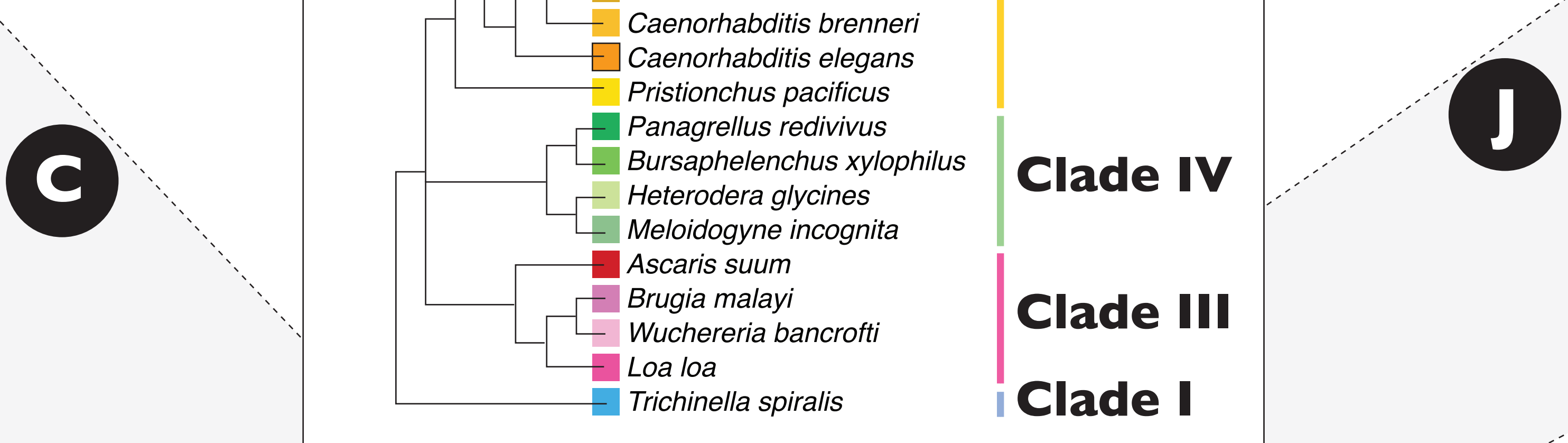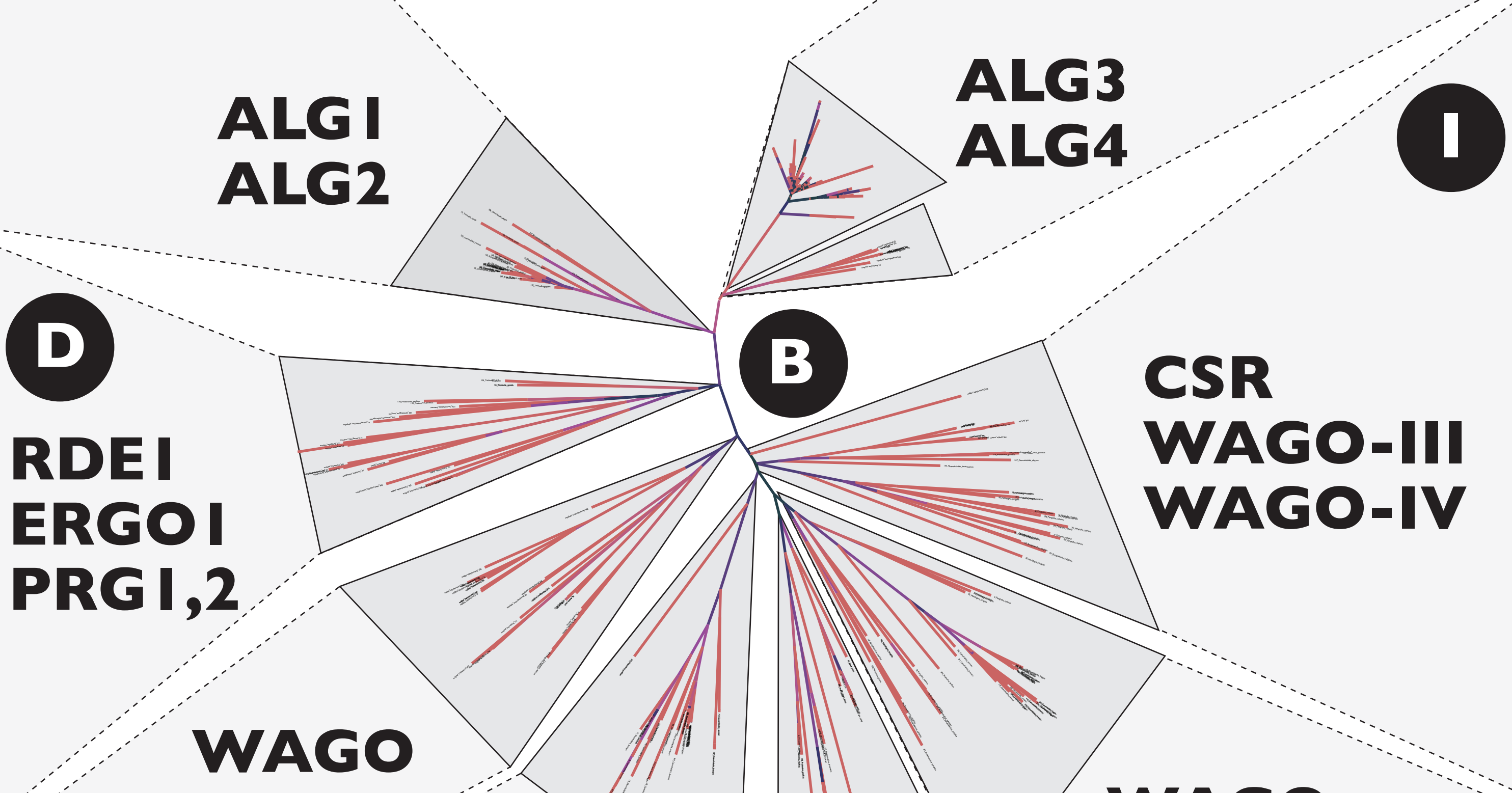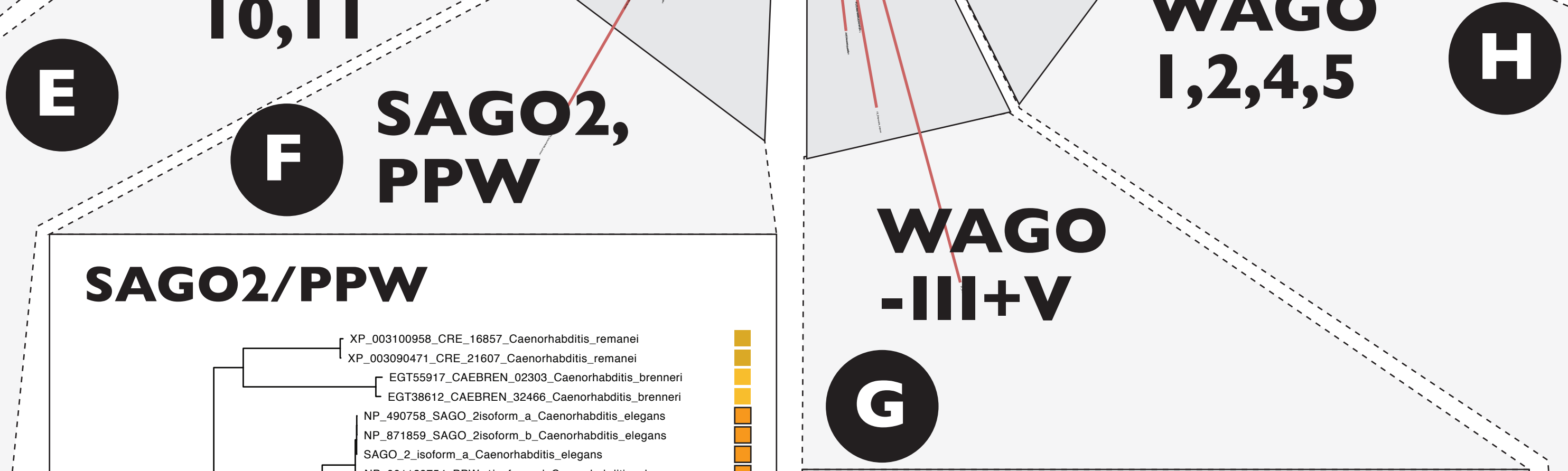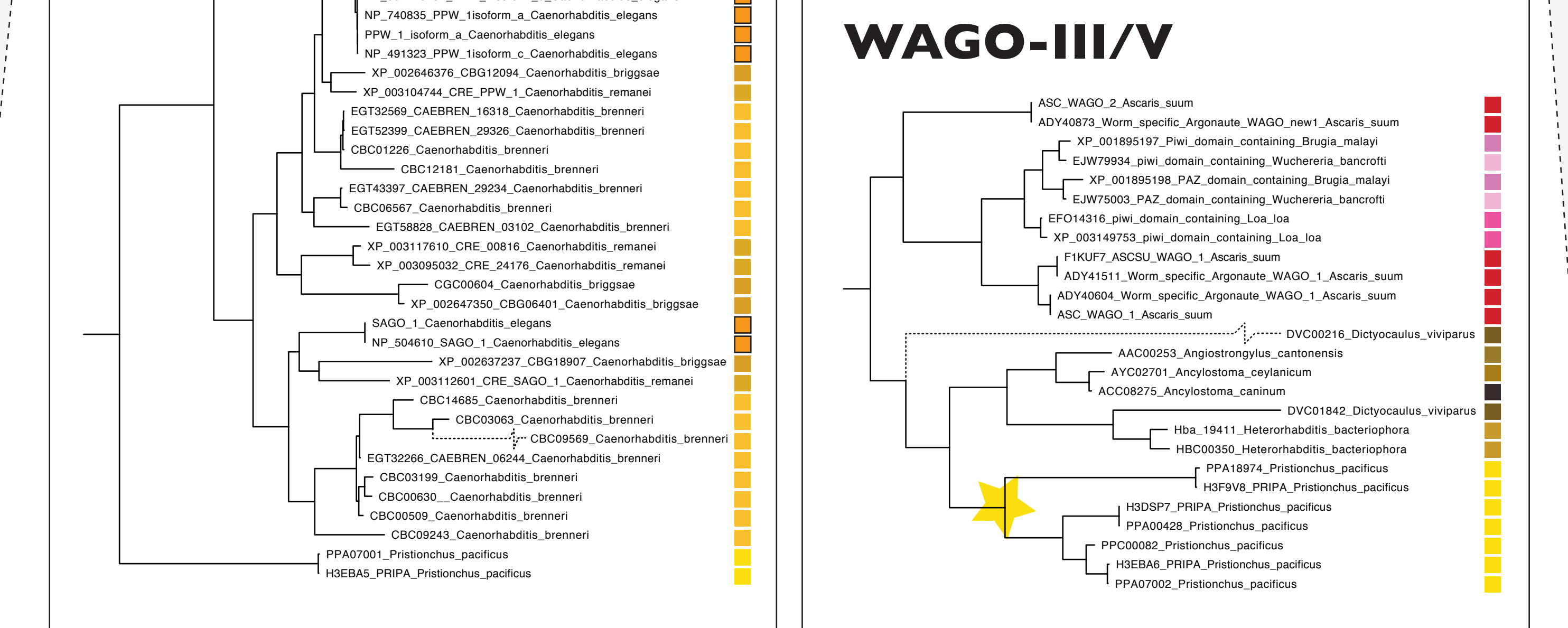

Supplement: Supplementary data [file bst0410881add.pdf]
